# Extracellular volume and left ventricular hypertrophy by cardiac magnetic resonance are independent predictors of cardiovascular outcome in obesity

**DOI:** 10.1038/s41598-022-23672-1

**Published:** 2022-11-05

**Authors:** Panuwat Lertlaksameewilai, Thammarak Songsangjinda, Yodying Kaolawanich, Ahthit Yindeengam, Rungroj Krittayaphong

**Affiliations:** Division of Cardiology, Department of Medicine, Faculty of Medicine Siriraj Hospital, Mahidol University, 2 Wanglang Road, Bangkoknoi, Bangkok, 10700 Thailand

**Keywords:** Cardiology, Obesity

## Abstract

This retrospective cohort study investigated for association between increased extracellular volume (ECV) and left ventricular hypertrophy (LVH) by cardiac magnetic resonance (CMR) and cardiovascular composite outcomes in obesity. Native T1 was measured at the ventricular septum. ECV was calculated from native and post-contrast T1 and hematocrit. Cardiovascular (CV) composite outcomes included acute myocardial infarction, unstable angina requiring hospitalization, myocardial revascularization (excluding early revascularization), heart failure, and CV death. A total of 456 patients with a mean follow-up of 2.1 ± 0.4 years were enrolled. LGE and LVH was detected in 30.5% and 9.2%. 107 patients (23.5%) had the composite outcomes. Multivariable analysis revealed that LGE, LVH, and high ECV as independent predictors for cardiovascular composite outcomes The event rate in the LVH and high ECV, the LVH alone, the high ECV alone, and the no-LVH with lower ECV group was 57.1%, 38.1%, 32.6%, and 17.7%, respectively. Assessment of incremental prognostic value by comparing global chi-square showed that high ECV had additional prognostic value on top of LGE, and LVH. LVH and high ECV are independent predictors of CV composite outcomes in obesity. This is the first study that demonstrate the prognostic value of ECV in obese population.

## Introduction

Obesity has become a highly and globally prevalent health crisis that increases the risk of cardiovascular disease^1^. Obesity is also an important component of metabolic syndrome, which promotes the development of cardiovascular disease and increases mortality^2^. In 2000 the Western Pacific Regional Office (WPRO) of World Health Organization (WHO) proposed an new definition of overweight (body mass index [BMI] 23.0–24.9) and obesity (BMI ≥ 25.0) for Asian populations^3^. At any BMI above 25 kg/m^2^, mortality risk is higher in Asian population compared to Caucasians^4^. Left ventricular hypertrophy (LVH) is also correlated with obesity^5^ via the mechanism of cardiac remodeling. The first mechanism is hemodynamic related to a volume overload condition. The second mechanism is mediated by a release of inflammatory cytokines and accumulation of adipose tissue in the myocardium. Both mechanisms can lead to subsequent myocardial fibrosis and LVH^6,7^. LVH and obesity have both been shown to be associated with many cardiovascular diseases, especially coronary artery disease (CAD) and heart failure (HF)^8,9^. Increased body mass index (BMI) was reported to be associated with ischemic cardiac events in patients with CAD^10^.

Cardiovascular magnetic resonance (CMR) demonstrates tissue property via longitudinal (spin–lattice) relaxation time to generate native T1 mapping. Myocardial native T1 lengthens correspondingly with interstitial expansion caused by edema, infarction, amyloid infiltration, and fibrosis. Conversely, native T1 shortens in the presence of fat and iron accumulation^11^. Native T1 mapping by CMR does not require the use of a contrast agent. Therefore, native T1 mapping can serve as a simple and non-invasive discriminator of cardiac muscle health and disease even in patients with impaired renal function who have contraindication for contrast agents. Obesity patients who have developed interstitial expansion have a higher myocardial native T1 values compared to normal-weight individuals^12^. Furthermore, extracellular volume (ECV) which represents the expansion of extracellular matrix can also be derived from the native- and post-contrast T1 mapping by quantifying the relative change in tissue T1 time to the blood pool following gadolinium contrast injection^13^. The consensus statement by the Society for Cardiovascular Magnetic Resonance (SCMR) endorsed by the European Association for Cardiovascular Imaging (EACVI) 2017 recommended routine assessment of ECV may be reasonable in patients receiving gadolinium-based contrast agents^13^.

The aim of this study was to determine whether increased ECV and LVH are risk predictors of cardiovascular composite outcomes in obesity.

## Results

### Patient characteristics and clinical outcomes

**Figure 1:**
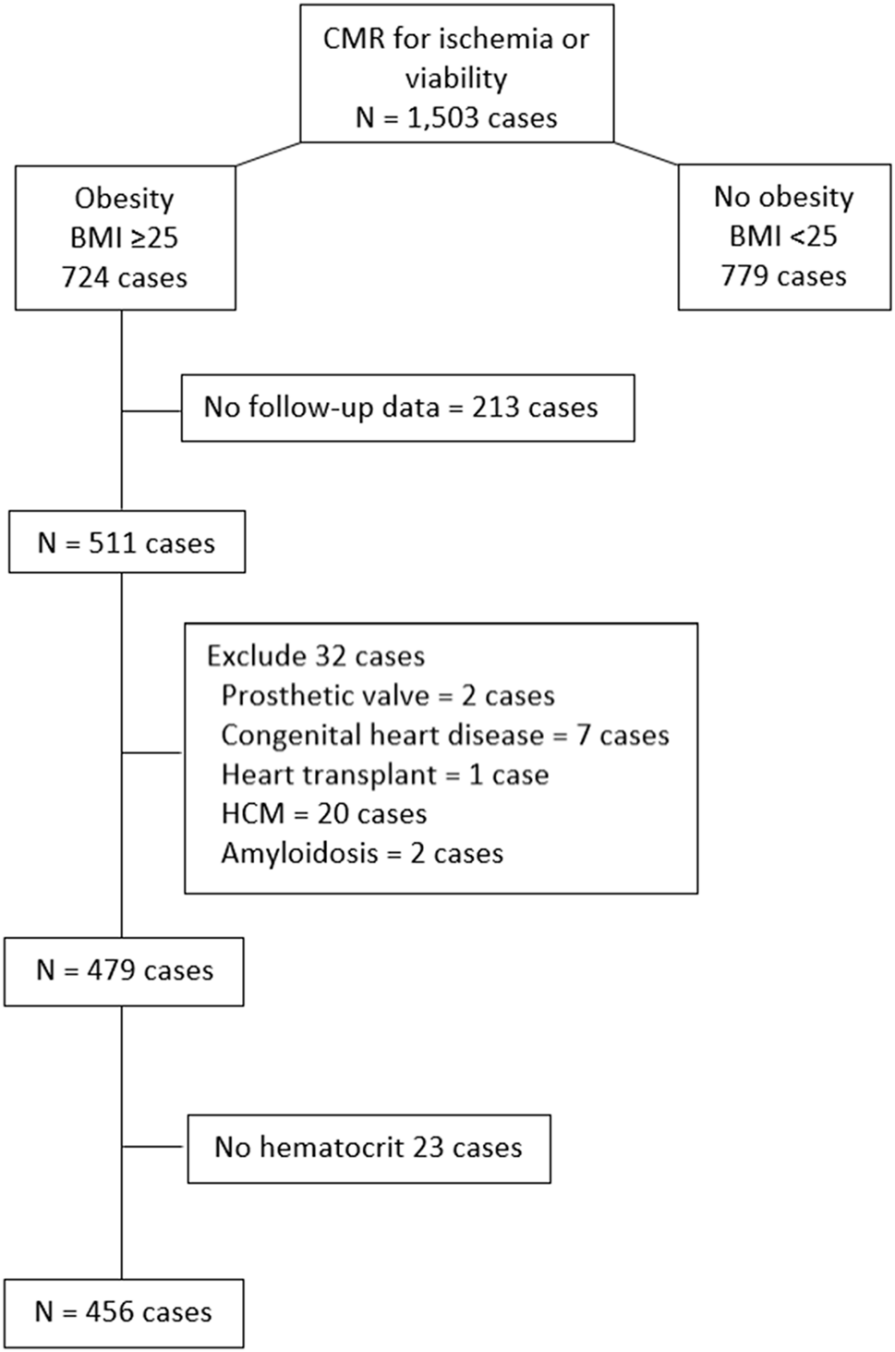
Flow diagram of the patient enrollment process.

Of the 1,503 subjects that underwent CMR during the study period, 456 subjects fulfilled all of the inclusion criteria and none of the exclusion criteria, so their data were included in the final analysis. The mean age of all patients was 70.1 ± 11.2 years, and 48.0% were male. Average BMI was 28.9 ± 3.6 kg/m^2^. A flow diagram describing the patient enrollment process is shown in Fig. 1. The reasons for CMR were the evaluation of stress myocardial perfusion in 98% of cases. Over a mean follow-up duration of 2.09 ± 0.43 years (median and interquartile range = 2.09 [1.87, 2.40] years), 107 patients (23.5%) had experienced the composite outcomes. The baseline characteristics of patients with and without clinical outcomes are shown in Table 1. The most common underlying condition was hypertension (89.7%), followed by dyslipidemia (82.5%). Thirty-seven patients (8.1%) had atrial fibrillation. Medication data were as follows: beta-blockers 335 (73.5%), calcium channel blockers 237 (52.0%), nitrates 143 (31.4%), statins 379 (83.1%), renin-angiotensin blockers 275 (60.3%), mineralocorticoid receptor antagonists 41 (9.0%), hydralazine 55 (12.1%), thiazide 79 (17.3%), antihyperglycemic agents 202 (44.3%), antiplatelets 311 (68.2%). Detail of cardiovascular outcomes that occurred during follow-up are shown in Supplementary Table 1.

**Table 1:** Baseline characteristics of the study population and comparison between those with and without the composite cardiovascular (CV) outcomes.

Factors	All patients (n = 456)	No CV outcome (n = 349)	CV outcomes (n = 107)	*p*-value
Male gender	219 (48.0%)	154 (44.1%)	65 (60.7%)	**0.003**
Age (yr)	70.1 ± 11.2	70.2 ± 11.2	69.8 ± 11.3	0.718
Body mass index (kg/m^2^)	28.9 ± 3.6	29.1 ± 3.7	28.3 ± 3.2	**0.056**
CAD risk factors	449 (98.5%)	342 (98.0%)	107 (100.0%)	0.207
Smoking	11 (2.4%)	6 (1.7%)	5 (4.7%)	**0.140**
Dyslipidemia	376 (82.5%)	288 (82.5%)	88 (82.2%)	0.947
Hypertension	409 (89.7%)	310 (88.8%)	99 (92.5%)	0.271
Diabetes	216 (47.4%)	161 (46.1%)	55 (51.4%)	0.340
History of CAD by coronary angiogram	117 (25.7%)	82 (23.5%)	35 (32.7%)	**0.056**
History of ACS	40 (8.8%)	24 (6.9%)	16 (15.0%)	**0.010**
STEMI	17 (3.7%)	11 (3.2%)	6 (5.6%)	0.249
NSTEMI	14 (3.1%)	7 (2.0%)	7 (6.5%)	**0.026**
UA	9 (2.0%)	6 (1.7%)	3 (2.8%)	0.444
CKD	255 (55.9%)	179 (51.3%)	76 (71.0%)	** < 0.001**
LVEF	66.8 ± 15.8	68.6 ± 14.3	61.0 ± 19.0	** < 0.001**
LVEF < 50%	69 (15.1%)	39 (11.2%)	30 (28.0%)	** < 0.001**
LV mass index (g/m^2^)	46.0 ± 17.1	43.7 ± 15.0	53.5 ± 21.1	** < 0.001**
LVH	42 (9.2%)	22 (6.3%)	20 (18.7%)	** < 0.001**
LGE present	139 (30.5%)	83 (23.8%)	56 (52.3%)	** < 0.001**
ECV (%)	28.8 ± 5.2	28.4 ± 5.2	30.0 ± 5.2	**0.006**

Data are presented as number and percentage or mean ± standard deviation.

A *p*-value < 0.05 indicates statistical significance (bold).

CAD, coronary artery disease; ACS, acute coronary syndrome; STEMI, ST-segment elevation myocardial infarction; NSTEMI, non-ST segment elevation myocardial infarction; UA; unstable angina; CKD, chronic kidney disease; CMR, cardiac magnetic resonance; LVEF, left ventricular ejection fraction; LVH, left ventricular hypertrophy; LGE, late gadolinium enhancement; ECV, extracellular volume.

### CMR findings

Baseline CMR characteristics of the whole population are shown in Supplementary Table 2. The prevalence of LVH was 9.2% (42/456). Patients with LVH were significantly older than no-LVH group (70.7 ± 10.7 vs. 64.6 ± 14.3 years; *p* = 0.010). Patients with LVH had a significantly higher native T1 and ECV compared to those without LVH (1349.5 ± 68.6 vs. 1316.4 ± 60.0; *p* = 0.001 and 31.1 ± 5.8 vs. 28.6 ± 5.1; *p* = 0.003, respectively). LGE was detected in 139 cases (30.5%); 27.2% were ischemic in origin, and 2.6% were non-ischemic and 0.7% were combined. The differences in CMR parameters between patients with and without cardiovascular outcomes are shown in Table 1.

### Univariate and multivariate analysis

Univariate and multivariate Cox proportional hazard model analysis was performed to identify factors that predict composite outcomes (Table 2). The variables that were included in the univariate and multivariate model were sex, age, smoking, dyslipidemia, hypertension, diabetes, history of CAD documented by coronary angiogram, history of acute coronary syndrome (ACS), chronic kidney disease (CKD) by estimated glomerular filtration rate (eGFR) less than 60 ml/min/1.73 m^2^ by Chronic Kidney Disease Epidemiology Collaboration (CKD-EPI) formula, low LVEF, LVH, LGE, and high ECV (top quartile). Multivariable analysis revealed that LGE, LVH, and high ECV as independent predictors for cardiovascular composite outcomes with the hazard ratio (HR), 95% confidence interval (CI) and p-value of 2.57 (1.75–3.80), *p* < 0.001, 1.87 (1.13–3.10), *p* = 0.015, and 1.70 (1.14–2.55), *p* = 0.009 respectively (Table 2). The incidence rates per 100 person-years of clinical outcomes in patients with and without LVH, and with and without high ECV are shown in Supplementary Table 3.

**Table 2:** Univariate and multivariate analyses for factors predicting the composite cardiovascular outcomes.

Factors	Univariate analysis		Multivariate analysis	
	HR (95% CI)	p	HR (95% CI)	p
Male	1.83 (1.24–2.70)	**0.002**	1.24 (0.78–1.98)	0.360
Age ≥ 65 yr	0.91 (0.60–1.37)	0.636	0.90 (0.57–1.42)	0.636
**CAD risk factors**				
Smoking	2.64 (1.08–6.50)	**0.034**	1.45 (0.57–3.72)	0.437
Dyslipidemia	0.96 (0.58–1.58)	0.869	0.89 (0.53–1.48)	0.650
Hypertension	1.40 (0.68–2.88)	0.357	1.02 (0.48–2.17)	0.952
Diabetes	1.17 (0.80–1.71)	0.414	1.13 (0.76–1.69)	0.539
History of CAD by coronary angiogram	1.44 (0.96–2.15)	** < 0.001**	1.40 (0.86–2.28)	0.179
History of prior ACS	1.95 (1.15–3.33)	**0.014**	1.34 (0.73–2.47)	0.344
CKD	2.06 (1.36–3.3)	**0.001**	1.48 (0.92–2.40)	0.108
LVEF < 50%	2.50 (1.64–3.81)	** < 0.001**	1.03 (0.61–1.74)	0.915
LVH	2.70 (1.66–4.39)	** < 0.001**	1.87 (1.13–3.10)	**0.015**
LGE present	2.97 (2.03–4.34)	** < 0.001**	2.57 (1.75–3.80)	** < 0.001**
ECV ≥ 30.8%*	2.16 (1.46–3.19)	** < 0.001**	1.70 (1.14–2.55)	**0.009**

A *p*-value < 0.05 indicates statistical significance (bold).

HR, hazard ratio; CI, confidence interval; CAD, coronary artery disease; ACS, acute coronary syndrome; CKD, chronic kidney disease; LVEF, left ventricular ejection fraction; LVH, left ventricular hypertrophy; LGE, late gadolinium enhancement; ECV, extracellular volume.

*Top quartile.

### Survival analysis

**Figure 2:**
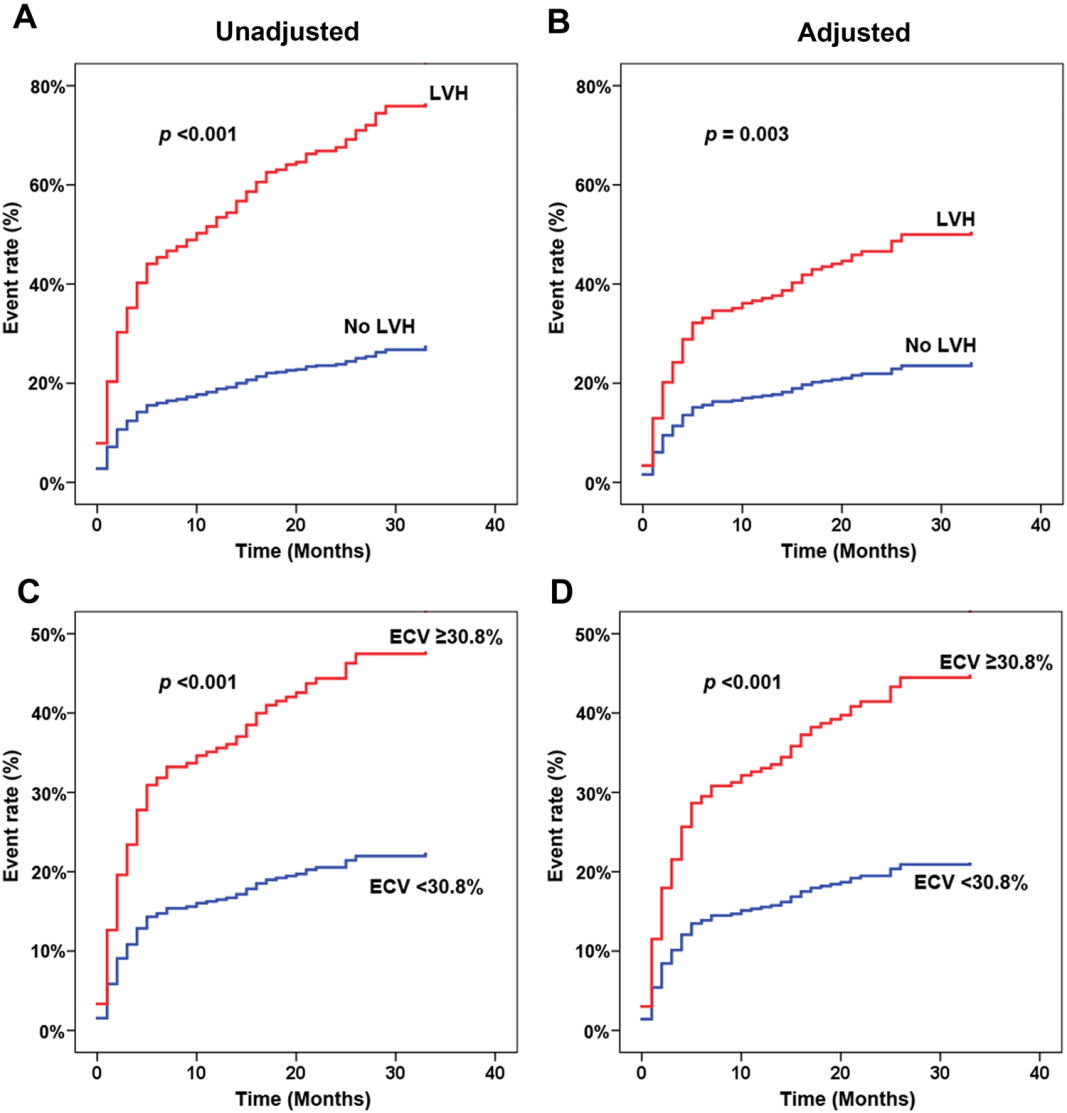
Cumulative incidence of cardiovascular event rate compared between LV hypertrophy (LVH) and no LVH: (**A**) unadjusted, (**B**) adjusted. Cumulative incidence of cardiovascular event rate compared between extracellular volume (ECV) < 30.8% and ECV ≥ 30.8%: (**C**) unadjusted, (**D**) adjusted.

Figure 2 demonstrates the unadjusted and adjusted hazard graphs of patients with and without LVH (Fig. 2A,B), and of patients with and without high ECV (Fig. 2C,D). Significant differences were observed for both unadjusted and adjusted models for LVH versus no-LVH, and for the top quartile ECV (≥ 30.8%) compared to the rest of study population. The confounders that were applied in the adjusted model were sex, smoking, hypertension, history of CAD, ACS, chronic kidney disease by history or by eGFR less than 60 ml/min/1.73 m^2^, and low LVEF. The hazard graphs of LVH versus no LVH, and high ECV versus the rest became more clearly separated as the follow-up time increased.

### Effect of combined LVH and increased ECV

**Figure 3:**
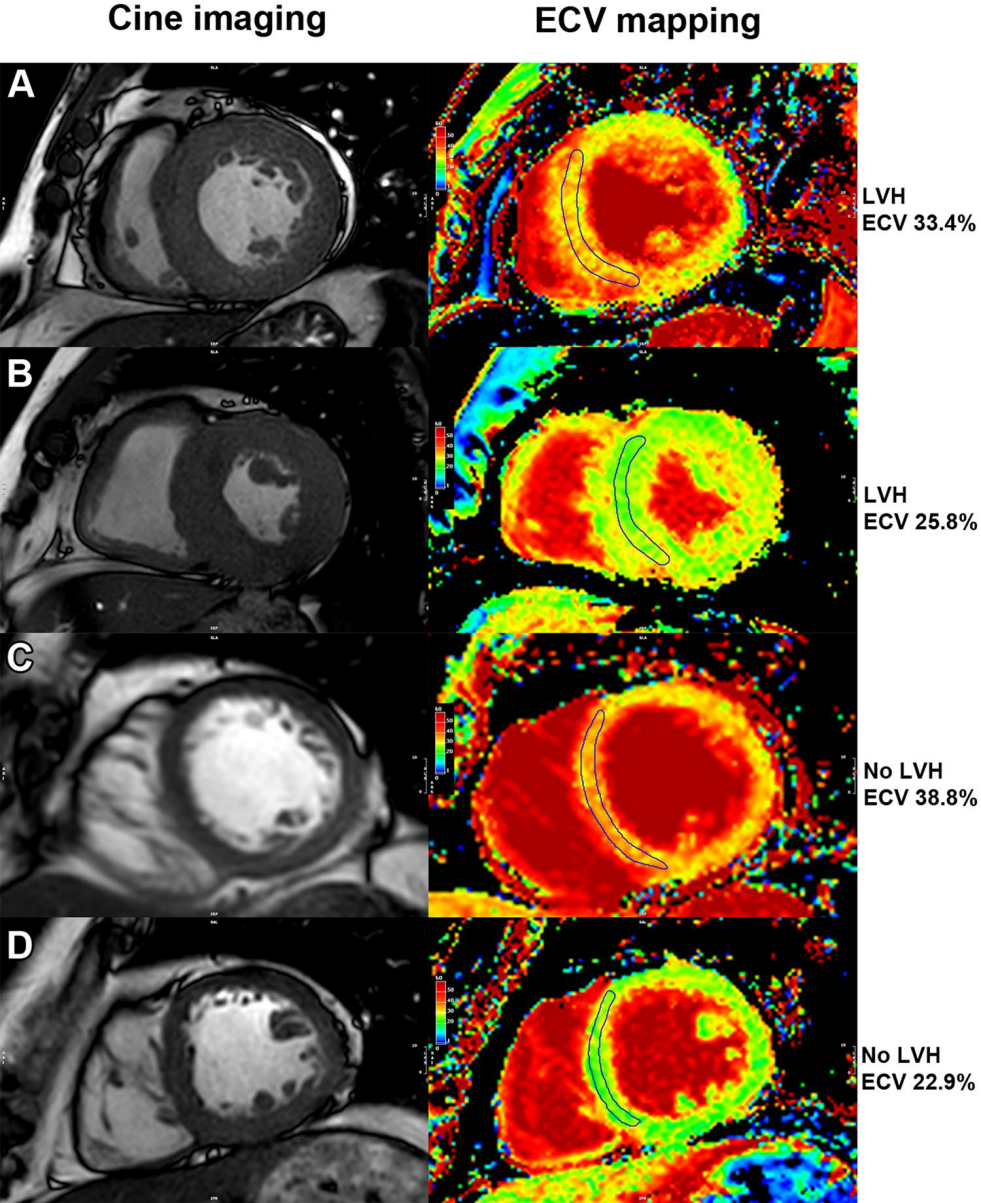
Cine images (left) and extracellular volume (ECV) mapping (right) of patients who had left ventricular hypertrophy (LVH) and high ECV (**A**), LVH and lower ECV (**B**), high ECV without LVH (**C**), and lower ECV without LVH (**D**).

**Figure 4:**
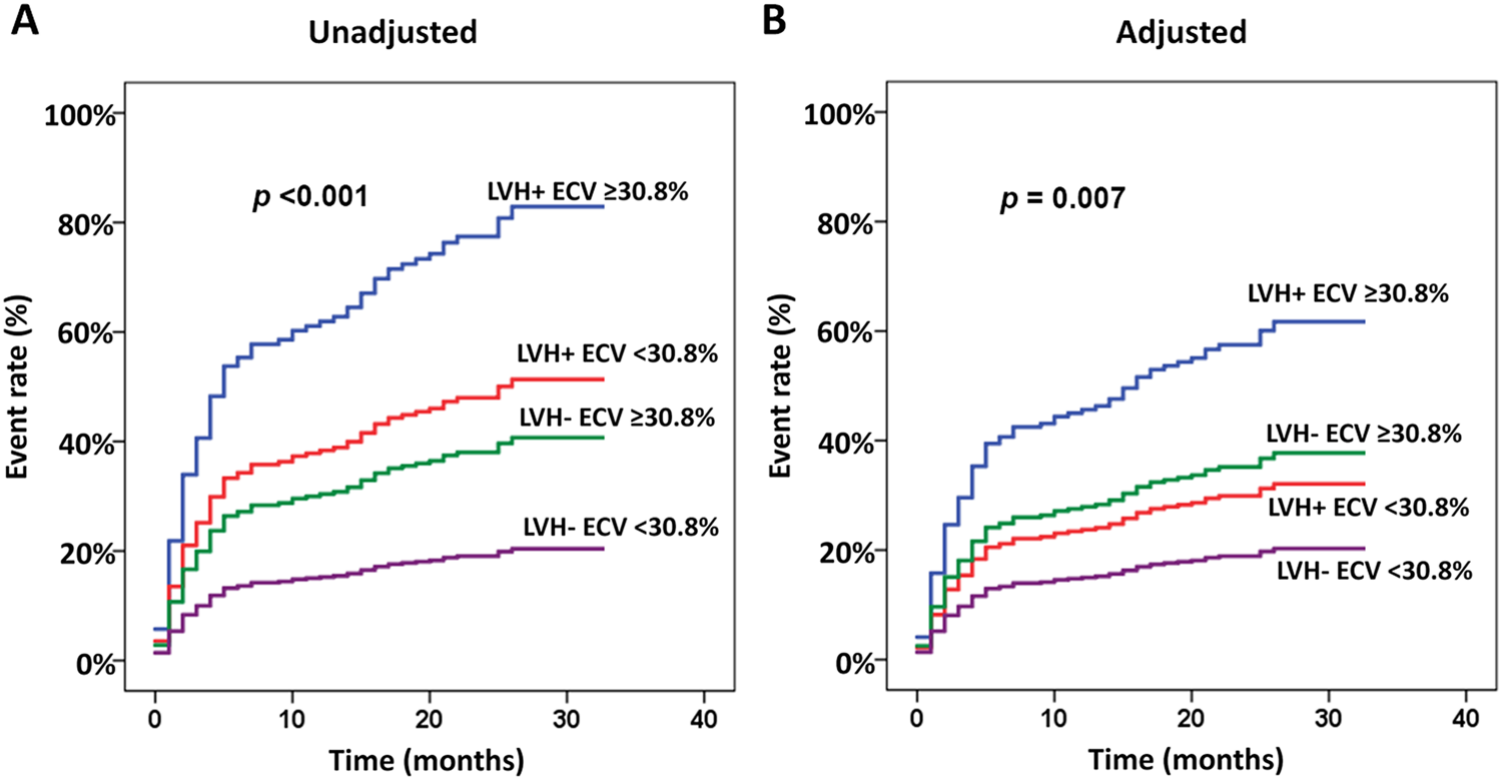
Cumulative incidence of cardiovascular event rate compared among LV hypertrophy (LVH) with extracellular volume (ECV) ≥ 30.8% (blue line), LVH with ECV < 30.8% (red line), no-LVH with ECV ≥ 30.8% (green line), and no-LVH with ECV < 30.8% (purple line): (**A**) unadjusted, (**B**) adjusted.

We explored the relationship between LVH and ECV for the prediction of composite outcomes. Among 42 patients with LVH, 21 (50%) had high ECV defined as ECV in the top quartile, whereas only 92 out of 414 patients without LVH (22.2%) had high ECV. Since both LVH and high ECV remained in the final multivariate analysis, we classified patients into the following 4 groups: group 1—LVH and high ECV (n = 21), group 2 – LVH alone (n = 21), group 3 – high ECV alone (n = 92), and group 4 – no-LVH with lower ECV (n = 322). Examples of cine images and ECV mapping of each group are shown in Fig. 3. The result showed that 12 patients (57.1%) in group 1, 8 patients (38.1%) in group 2, 30 patients (32.6%) in group 3, and 57 patients (17.7%) in group 4 developed a clinical event during the follow-up (*p* < 0.001) (Fig. 4A). After adjustment of potential confounders, the significant differences among groups remained (Fig. 4B).

**Figure 5:**
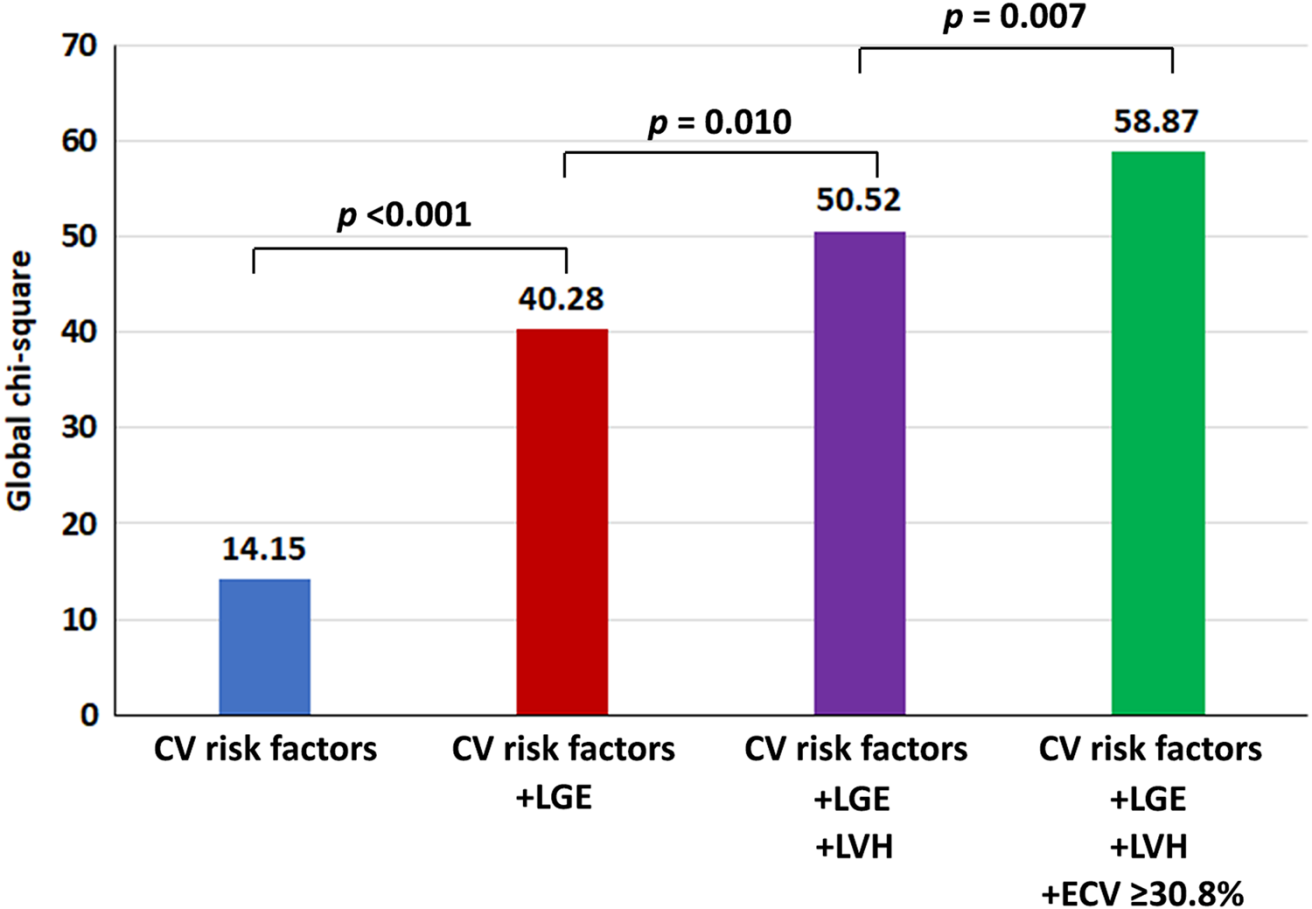
Incremental prognostic value shown as global chi-square values on y-axis of cardiovascular (CV) risk factors, late gadolinium enhancement (LGE), left ventricular hypertrophy (LVH), and extracellular volume fraction (ECV) for composite outcomes.

The incremental prognostic value was assessed using baseline cardiovascular risk factors (i.e., age, sex, diabetes, hypertension, dyslipidemia, and smoking), the 3 variables that remained in the multivariate model, i.e., LGE, LVH and high ECV. The global chi-square was significantly higher at each step of addition of these variables starting from CV risk factors, LGE, LVH and ECV. This finding indicates an incremental prognostic value of LGE, LVH and ECV in this study population (Fig. 5).

### Sensitivity analysis

**Figure 6:**
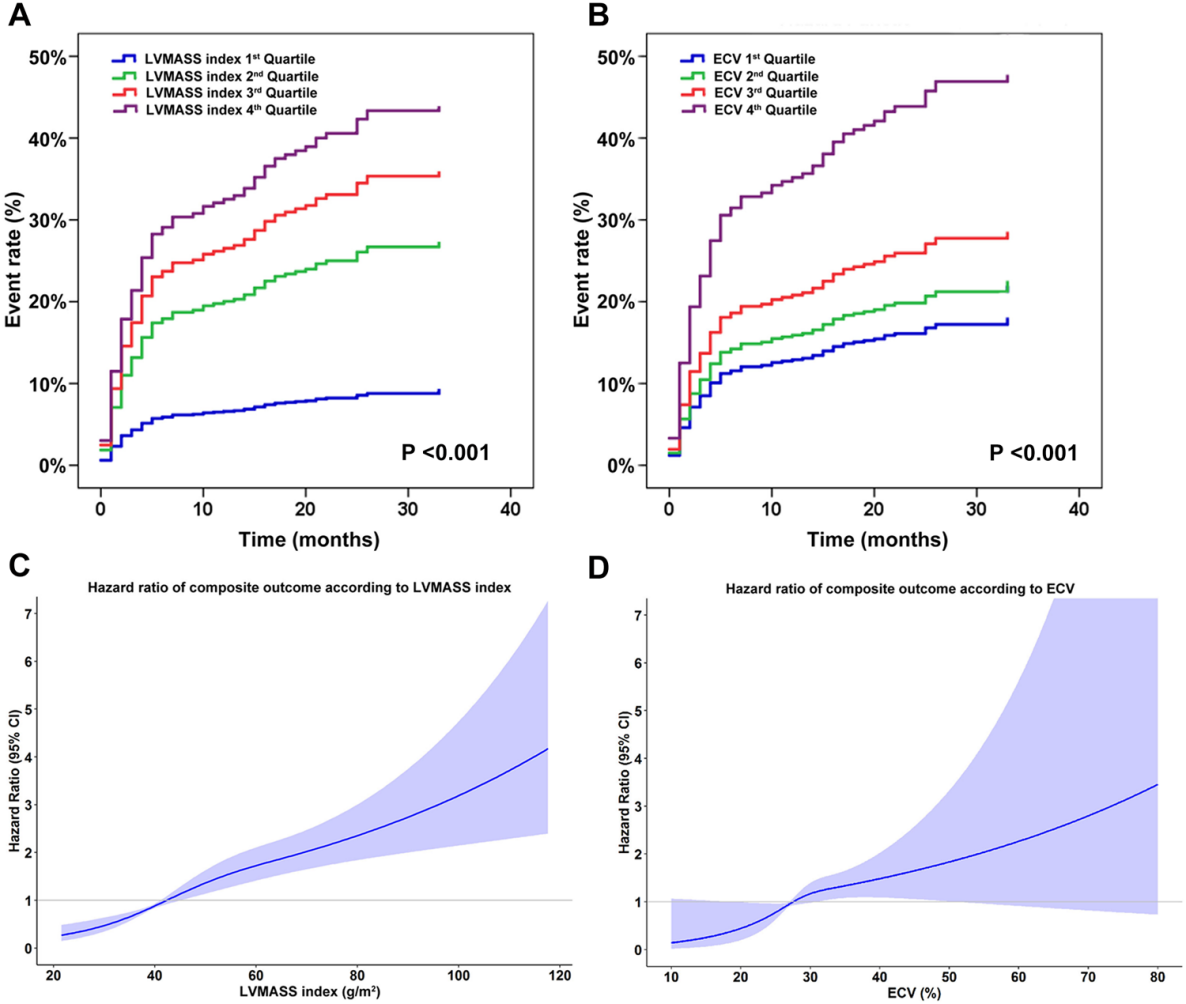
Sensitivity analysis. (**A** and **B**) The effect of left ventricular mass (LVMASS) index and extracellular volume (ECV) on composite outcomes when the variables were subdivided into quartiles. (**C** and **D**) Cubic spline graphs showing the effect of LVMASS index and ECV on composite outcomes when the variables were treated as continuous variables.

Sensitivity analysis was performed by comparing the effect of left ventricular (LV) mass index and ECV stratified into quartiles on clinical outcomes using survival analysis. The results are shown in Fig. 6A,B. Increased LV mass index was significantly associated with an increase in the rate of clinical outcomes (*p* < 0.001). LV mass index in the 4th, 3rd, 2nd quartiles had a HR (95% CI) of 4.94 (2.39–10.20), 4.03 (1.93–8.42), and 3.04 (1.43–6.49), respectively, compared to those in the 1st quartile. In a similar trend, ECV in the 4th, 3rd, 2nd quartiles had a HR (95% CI) of 2.73 (1.55–4.79), 1.61 (0.88–2.96), and 1.23 (0.65–2.34), respectively, compared to those in the 1st quartile (*p* = 0.001). As shown in Fig. 6A,B, LV mass index significantly increased risk from the 2nd quartile compared to the 1st quartile, whereas ECV significantly increased risk only in the 4th quartile.

We also performed analysis by using the ECV cut-off from a receiver operating characteristic (ROC) curve to define high ECV. The best cut-off from the ROC curve analysis was 28.9%. When using this cut-off to classify high ECV, the results of univariate and multivariate analysis remained the same. High ECV using this cut-off significantly and independently predicted clinical outcomes (all *p* < 0.001). When we evaluated effect of combined LVH and high ECV status using the ROC curve cut-off to define high ECV, the results were similar to primary analysis. Group 1 (LVH with high ECV) had the greatest risk of clinical outcomes with a HR (95% CI) of 4.65 (2.61–8.30). Group 2 (LVH alone) and group 3 (high ECV alone) had a HR (95% CI) of 1.75 (0.63–4.88) and 1.75 (1.15–2.66), respectively.

Additional sensitivity analysis was performed to assess the effect of LV mass index and ECV on clinical outcomes by treating each variable as continuous data. A cubic spline graph was generated to assess the effect of the LV mass index and ECV on the hazard ratio (Fig. 6C,D). The results of that analysis showed that the hazard ratio of clinical outcomes increased as the LV mass index and ECV increased, which supports the results of our primary analysis.

We tested the relationship between ECV and hypertension and CKD, and between LVH and hypertension and CKD. The results showed that high ECV and LVH is more common in patients with hypertension (high ECV 26.2% in hypertension vs 12.8% in non-hypertension, *p* = 0.044, LVH 10.0% in hypertension vs 0% in non-hypertension, *p* = 0.021), and CKD (high ECV 30.6% in CKD vs 17.4% in non-CKD, *p* = 0.001, LVH 13.3% in CKD vs 4.0% in non-CKD, *p* = 0.001). Then, we analysed the interaction of hypertension and CKD on the predictive values of ECV and LVH on clinical outcomes. We demonstrated that there were no significant interactions which meant that the predictive values of ECV and LVH were not influenced by the presence or absence of CKD or hypertension. The interaction test p-value was 0.606 for CKD and LVH, 0.734 for CKD and ECV, 0.867 for hypertension and ECV. Interaction test cannot be performed for hypertension and LVH since all patients with LVH had hypertension.

We added analysis of LV phenotypes and relation of LV phenotypes and ECV and clinical outcomes. Patients were classified into 4 groups according to LV mass: normal 388 (85.1%), concentric remodeling 26 (5.7%), concentric hypertrophy 14 (3.1%), and eccentric hypertrophy 28 (6.1%). Patients with eccentric hypertrophy had the highest ECV which was significantly different compared to normal whereas concentric remodeling and concentric hypertrophy had ECV values in the middle between normal and eccentric hypertrophy. The ANOVA test showed p-value 0.08 for the difference between groups. Cox-proportional Hazard model showed that LV phenotypes can predict the composite outcome with a p-value < 0.001 which was mainly due to the signiticant difference of eccentric hypertrophy compared to normal [HR 3.34 (1.95–5.71), p < 0.001]. The HR and 95% CI of concentric remodeling and concentric hypertrophy were 1.1 (0.48–2.52), *p* = 0.822 and 1.56 (0.57–4.25), *p* = 0.388, respectively. There was no significant interaction for the inflence of LV phenotypes on the effect of ECV on clinical outcomes.

Lastly, we performed sensitivity analysis of univariate and multivariate analysis of clinical outcomes by using continuous data for age, eGFR, LVEF, and LV mass index. The results are shown in Supplementary Table 4 which demonstrated that the 3 variables that were significant predictors on multivariate analysis in Table 2 remained significant determinants of clinical outcomes.

## Discussion

In this study, we assessed the effect of LVH and ECV (quantified by CMR) and cardiovascular composite outcomes over a mean follow-up of 2.1 years. LVH and high ECV were found to be independent predictors of cardiovascular composite outcomes (HR 2.13 for both). Kaplan–Meier analysis demonstrated that the risk of cardiovascular composite outcomes was highest in patients with LVH combined with high ECV, followed by each of those 2 factors alone.

Data from the Multi-Ethnic Study of Atherosclerosis (MESA) study^14^ showed a relationship between obesity-related LVH and cardiovascular composite outcomes similar to that found in our study (HR 1.58, 96% CI 1.15–2.18; *p* = 0.005 vs. HR 2.13, 95% CI 1.29–3.49; *p* = 0.003, respectively). The findings from our study support the result of a previous study that LVH assessed by CMR indicated an increased risk of cardiovascular events^15^. However, the incremental value of ECV assessment when combined with LVH shown in our study provides additional insight.

Myocardium with fibrosis has a higher ECV and higher native T1, which corresponds with an increase in extracellular volume^11^. From a previous systematic review and meta-analysis, ECV appeared to have more consistent data for the prediction of clinical outcomes compared to native T1 values^16^. An excessive accumulation of extracellular matrix leading to myocardial fibrosis, affects both the structural and electrical properties of cardiac myocytes^17,18^. There have been a conflicting results for the influence of obesity on ECV changes. Some studies reported an increase in ECV in obesity^12,19^ whereas the other study found that ECV in patients with obesity was reduced^20^. The result of our study indicted that obesity with a higher ECV had an increased risk of adverse cardiac outcome especially when combined with LVH.

An increased ECV in patients with LVH confirms that the remodeling process resulting in LVH is associated with myocardial fibrosis^11^, and associated with cardiovascular events^17^. In our study, patients with LVH or high ECV had an increased risk of cardiovascular events. Although LVH can cause myocardial fibrosis resulting in an increased ECV, LVH and ECV were both individually identified as independent risk factors for cardiovascular events. Moreover, we found that patients with both LVH and high ECV had the highest risk of cardiovascular events. Patients with only LVH or only high ECV also had an increased risk of cardiovascular events compared to those without LVH or high ECV, but the risk of patients who had only a single factor is lower than in those with combined factors. As a result, patients with both LVH and high ECV had the worst prognosis of cardiovascular outcomes.

Since echocardiogram is usually the initial investigation, we think that the results from our study can be applied for echocardiogram. LVH can be reliably assessed by echocardiogram and can be used as a prognostic marker^21^. LV phenotypes that we performed additional analysis can also be applied for echocardiogram^22^.

There are several ways to treat obesity, including diet control, exercise, medications, and bariatric surgery. From a previous systematic review, weight-loss via bariatric surgery can reduce the risk of cardiovascular diseases, decrease the size of left ventricular mass in patients with LVH, and improve diastolic function^23^. The Losartan Intervention For Endpoint Reduction (LIFE) study reported that a decrease in the size of a left ventricular mass in LVH patients was associated with reduced incidence of sudden cardiac death^24^. Future studies are needed to explore the effect of the treatment for obesity on changes in LVH and ECV.

### Study limitations

This study has some limitations that need to be disclosed. First, this was a single-center study. As such, a multi-center study with a much larger study population is needed to confirm and perhaps broaden the findings of this study. Second, this study was based on data from patients who were referred for CMR for clinical purpose, most of whom were suspected CAD. There were 213 obese patients (25.6%) who were not included in the final analysis due to inadequate follow-up data– results may not reflect the real practice. Therefore, our results may not be generalizable to all obese population. Third, hematocrit values within 3 months of CMR might not represent the most current hematocrit for ECV calculation. Fourth, we aimed to study patients with obesity which is a specific group of population. However, the data showed that patients with obesity account for 48% of patients referred for CMR in our center, consistent with the obesity prevalence worldwide (approximately 40%)^25^ and in Thailand^26^. This population group has an increased risk of developing cardiovascular disease as well as coronary artery disease^27^ despite obesity paradox^28^ may be possible. Therefore, we did not analyse data in non-obesed population. The results of this study may not be generalized to other patient population. Lastly, native T1 and ECV were sampled at the septum based on the assumption that the septum could represent diffuse myocardial fibrosis with minimal susceptibility artifact from adjacent structures^13^. Even though the septum would not always represent the whole paramagnetic property of the entire myocardium, a recent consensus recommended measuring T1 at the septum of mid LV level as the standard measurement technique^13^. Moreover, the septum was shown to have the greatest precision, and minimized the effect of variations in regional T1 values due to artifact of the left ventricular free wall myocardium^29^.

## Conclusion

The results of this study revealed LVH and high ECV to both be independent predictors of cardiovascular composite outcomes in obesity. Combining LV mass and ECV data was also shown to have additive prognostic value for cardiovascular outcomes in patients with obesity.

## Methods

### Study design

This retrospective cohort study was approved by the Siriraj Institutional Review Board (SIRB) of the Faculty of Medicine Siriraj Hospital, Mahidol University, Bangkok, Thailand (COA no. 680/2020). The need for Informed consent was waived by the IRB of the Faculty of Medicine Siriraj Hospital due to retrospective nature of the study. All methods was conducted in accordance with the principles set forth in the Declaration of Helsinki and the International Conference on Harmonization for Good Clinical Practice Guidelines.

### Study population

We enrolled subjects who had to be at least 18 years of age, obese (body mass index [BMI] > 25 kg/m^2^ according to the WHO WPRO Asian definition criteria)^3,30^, who underwent clinical CMR at our center from July 2017 to September 2018 and were followed-up for at least 6 months. Subjects were excluded if they had a history of cardiovascular surgery except for coronary artery bypass surgery, congenital heart disease, hypertrophic cardiomyopathy, amyloidosis, or had inadequate follow-up data (> 6 months)—patients who did not have hematocrit values within 3 months before CMR were also excluded. Information of baseline and follow-up visits was collected from electronic-based medical records.

### Cardiac magnetic resonance (CMR) protocol

CMR was performed using an Ingenia 3.0 T MR system (Philips Healthcare, Best, the Netherlands). The CMR protocol included black blood axial images, steady-state free precession (SSFP) cine images of standard views, and native and post-contrast T1 mapping. T1 mapping was performed with breath-holding technique in mid-diastole using 5-(3)-3 modified Look-Locker inversion recovery (MOLLI) sequence^31^ in a single mid-ventricular short axis slice with repetition time (TR) 2.2 ms, echo time (TE) 1.8 ms, 8 different inversion time (TIs), matrix 152 × 150, field of view 300 × 300 mm^2^, flip angle 20°, sensitivity encoding (SENSE) 2, and slice thickness of 10 mm). For patients who underwent stress CMR, First-pass perfusion study was performed by an injection of 0.05 mmol/kg of gadolinium contrast.

Agent [gadoterate meglumine (Dotarem), gadobutrol (Gadovist), or gadopentetate dimeglumine (Magnevist)]. Another injection of 0.1 mmol/kg gadolinium was administered immediately after the acquisition of perfusion images. Late gadolinium enhancement (LGE) images covering the entire left ventricle were acquired approximately 10 min after the injection by the 3D segmented-gradientecho inversion-recovery sequence. A post-contrast T1 mapping was then performed. Parameters for cine images were repetition time/echo time/number of excitations 3.7/1.8/2, 390 × 312-mm field of view, 256 × 240 matrix, 1.52 × 1.21 reconstruction pixel, 8-mm slice thickness, and 70° flip angle. The LGE images were acquired with the use of 3-dimensional segmented-gradient-echo inversion- recovery sequence with echo time 1.25, repetition time 4.1, 15° flip angle, 303 × 384-mm field of view, 240 × 256 matrix, in-plane resolution 1.26 × 1.5 mm, slice thickness 8 mm, and 1.5 sensitivity encoding factor.

### Imaging assessment

Analysis of CMR images was performed on IntelliSpace Portal (ISP) software version 11.1 (Philips Healthcare, Best, the Netherlands). The endocardial and epicardial borders of all short-axis images were automatically detected and manually adjusted during end-diastole and end-systole. Left ventricular ejection fraction (LVEF) was calculated from the end-diastolic and end-systolic volume, and the result is presented as a percentage. Left ventricular (LV) mass was derived from the summation of mass during the end-diastole of all left ventricular short-axis slices and indexed by the body surface area (BSA). The definition of LVH was LV mass index greater than or equal to the 95th percentile of normal volunteers^32^, which was 77.9 g/m^2^ in men, and 60.8 g/m^2^ in women. LGE images were analyzed by visual assessment based on the consensus of 2 readers and were interpreted as ischemic or non-ischemic. For ischemic LGE, transmural extent of LGE was graded as subendocardial or transmural scar for each myocardial segment according to the recommendation of American Heart Association (AHA)^33^. The analysis was blinded to the patient’s name and functional images.

Native T1 mapping was derived by the exponential fitting curve of the inversion recovery. A region of interest (ROI) was manually drawn covering the entire septum of both native- and post-contrast T1 mapping, and the average value of T1 within the ROI was recorded. For patients who had myocardial scar at the septum, the ROI was selected from non-scar area which may be outside the septal region. According to the recommendation by society of cardiovascular magnetic resonance (SCMR)^13^, region of interest (ROI) for T1 mapping that was used to calculate ECV can be drawn at the septal segments or a complete single short-axis slice (usually a mid-ventricular slice). However, a single ROI drawn in the septum on mid-cavity short-axis maps is preferred to avoid lung, liver and veins as sources of susceptibility artifacts. In another review article^29^, the authors summarized that septal sampling has been shown to yield the greatest precision and minimizes the effect of considerable variations of regional T1 values caused by the artifact-prone left ventricular free wall myocardium^34^. Myocardial ECV was then calculated with the following formula^13^. The patient’s hematocrit within 3 months before CMR was used:

$${\text{ECV }}\left( {\text{\% }} \right) = \left( {100 - {\text{Hematocrit}}} \right) \times \left( {\frac{{\frac{1}{{{\text{Post contrast T}}1_{{\text{Myocardium }}} }} - \frac{1}{{{\text{Native T}}1_{{\text{Myocardium }}} }}}}{{\frac{1}{{{\text{Post contrast T}}1_{{\text{Blood }}} }} - \frac{1}{{{\text{Native T}}1_{{{\text{Blood}}}} }}}}} \right){ }$$

ECV of study subjects were considered high if those values fall within the top quartile of the study population. The analysis of LGE and T1 mapping were blinded to the patient’s name and functional images.

### Outcomes

Cardiovascular composite outcomes were defined as cardiovascular events consisting of acute myocardial infarction, unstable angina requiring hospitalization, coronary revascularization (exluding early revascularization within 3 months after CMR), heart failure, or cardiovascular death. Clinical outcomes were defined according to the 2014 recommendation of the American College of Cardiology (ACC) and AHA for key data elements and definitions for cardiovascular endpoint events in clinical trials^35^. For example, heart failure event was defined as a hospital admission or a presentation of the patient for an urgent, unscheduled clinic/office/emergency department visit, with a primary diagnosis of heart failure, whereby the patient exhibits new or worsening symptoms of heart failure on presentation, has objective evidence of new or worsening heart failure, and receives initiation or intensification of treatment specifically for heart failure. We collected clinical outcomes from clinical visits and medical records. The clinical outcomes were adjudicated by 2 cardiologists completely blinded to clinical and CMR data. Disagreement was solved by the 3rd cardiologist. Script telephone interview was also performed to collect data that might be missing from medical record.

### Statistical analysis

Continuous variables were compared by Student’s *t*-test for unpaired data, and are expressed as mean and standard deviation. Categorical variables were compared by chi-square test, and are presented as number and percentage. Univariate and multivariate Cox-proportional hazard model analysis was performed to identify factors that independently predict composite outcomes. Kaplan–Meier analysis and Cox regression were applied to assess the impact of LVH on cardiovascular outcomes. The log-rank test was used to analyze the difference between groups. The primary analysis was based on the aforementioned definition of LVH and high ECV. The incremental prognostic value of variables was performed based on variables that remain in the final model of multivariate analysis. The incremental values were assessed by considering these variables in hierarchical order and comparing the global chi-square derived from each hierarchical model. Sensitivity analysis was performed by (1) comparing the effect of LV mass index and ECV stratified into quartiles on clinical outcomes (2) using the ECV cut-off from the receiver operating characteristic (ROC) curve to define a high ECV, and (3) assessing the effect of the LV mass index and ECV on clinical outcomes by treating each variable as continuous data (4) studying the influence of hypertension and CKD on the prognostic value of LVH and ECV on clinical outcomes and (5) assessing the LV phenotypes^22^ and effect on ECV and outcomes. To that end, a cubic spline graph was generated to assess the effect of LV mass index and ECV on clinical outcomes. A *p*-value of < 0.05 was considered to be statistically significant.

## Supplementary Information

Supplementary Information.

## Data Availability

The dataset that was used to support the results and conclusion of this study are included within the manuscript. The additional data are available from corresponding author upon reasonable request.

## References

[1] Csige I (2018). The impact of obesity on the cardiovascular system. J. Diabetes Res..

[2] Grundy SM (2008). Metabolic syndrome pandemic. Arterioscler Thromb. Vasc. Biol..

[3] World Health Organization, R. O. F. T. W. P. W., *International Association for the Study of Obesity, International Obesity Task Force*. (Health Communications Australia Pty Ltd, 2000).

[4] Wen CP (2009). Are Asians at greater mortality risks for being overweight than Caucasians? Redefining obesity for Asians. Public Health Nutr..

[5] Lauer MS, Anderson KM, Kannel WB, Levy D (1991). The impact of obesity on left ventricular mass and geometry. The Framingham Heart Study. JAMA.

[6] Murdolo G (2015). Left ventricular hypertrophy and obesity: Only a matter of fat?. High Blood Press. Cardiovasc. Prev..

[7] Liu CY (2017). Association of elevated NT-proBNP with myocardial fibrosis in the multi-ethnic study of atherosclerosis (MESA). J. Am. Coll. Cardiol..

[8] Bogers RP (2007). Association of overweight with increased risk of coronary heart disease partly independent of blood pressure and cholesterol levels: A meta-analysis of 21 cohort studies including more than 300 000 persons. Arch. Intern. Med..

[9] Hoang K (2015). LV mass as a predictor of CVD events in older adults with and without metabolic syndrome and diabetes. JACC Cardiovasc. Imaging.

[10] Fihn SD (2012). 2012 ACCF/AHA/ACP/AATS/PCNA/SCAI/STS guideline for the diagnosis and management of patients with stable ischemic heart disease: a report of the American College of Cardiology Foundation/American Heart Association task force on practice guidelines, and the American College of Physicians, American Association for Thoracic Surgery, Preventive Cardiovascular Nurses Association, Society for Cardiovascular Angiography and Interventions, and Society of Thoracic Surgeons. Circulation.

[11] Radenkovic D, Weingartner S, Ricketts L, Moon JC, Captur G (2017). T1 mapping in cardiac MRI. Heart Fail. Rev..

[12] Homsi R (2019). Epicardial fat, left ventricular strain, and T1-relaxation times in obese individuals with a normal ejection fraction. Acta Radiol..

[13] Messroghli DR (2017). Clinical recommendations for cardiovascular magnetic resonance mapping of T1, T2, T2* and extracellular volume: A consensus statement by the Society for Cardiovascular Magnetic Resonance (SCMR) endorsed by the European Association for Cardiovascular Imaging (EACVI). J. Cardiovasc. Magn. Reson..

[14] Chirinos JA (2010). Left ventricular mass: Allometric scaling, normative values, effect of obesity, and prognostic performance. Hypertension.

[15] Kawel-Boehm N (2019). Left ventricular mass at MRI and long-term risk of cardiovascular events: The Multi-Ethnic Study of Atherosclerosis (MESA). Radiology.

[16] Zhuang B (2018). Prognostic value of T1 mapping and extracellular volume fraction in cardiovascular disease: A systematic review and meta-analysis. Heart Fail. Rev..

[17] Ambale-Venkatesh B (2019). Association of myocardial fibrosis and cardiovascular events: The multi-ethnic study of atherosclerosis. Eur. Heart J. Cardiovasc. Imaging.

[18] Schmidt A (2007). Infarct tissue heterogeneity by magnetic resonance imaging identifies enhanced cardiac arrhythmia susceptibility in patients with left ventricular dysfunction. Circulation.

[19] Shah RV (2013). Myocardial tissue remodeling in adolescent obesity. J. Am. Heart Assoc..

[20] van den Boomen M (2018). Native T1 reference values for nonischemic cardiomyopathies and populations with increased cardiovascular risk: A systematic review and meta-analysis. J. Magn. Reson. Imaging.

[21] Verdecchia P (1996). Prognostic value of left ventricular mass and geometry in systemic hypertension with left ventricular hypertrophy. Am. J. Cardiol..

[22] Rodrigues JC (2016). Comprehensive characterisation of hypertensive heart disease left ventricular phenotypes. Heart.

[23] Vest AR, Heneghan HM, Agarwal S, Schauer PR, Young JB (2012). Bariatric surgery and cardiovascular outcomes: A systematic review. Heart.

[24] Wachtell K (2007). Regression of electrocardiographic left ventricular hypertrophy during antihypertensive therapy and reduction in sudden cardiac death: The LIFE Study. Circulation.

[25] Stierman, B. *et al.**National Health and Nutrition Examination Survey 2017–March 2020 Prepandemic Data Files Development of Files and Prevalence Estimates for Selected Health Outcomes* (2021). 10.15620/cdc:106273 PMC11513744 39380201

[26] Sakboonyarat B (2020). Trends, prevalence and associated factors of obesity among adults in a rural community in Thailand: Serial cross-sectional surveys, 2012 and 2018. BMC Public Health.

[27] Powell-Wiley TM (2021). Obesity and cardiovascular disease: A scientific statement from the American Heart Association. Circulation.

[28] Carbone S (2019). Obesity paradox in cardiovascular disease: Where do we stand?. Vasc. Health Risk Manag..

[29] Puntmann VO, Peker E, Chandrashekhar Y, Nagel E (2016). T1 Mapping in characterizing myocardial disease: A comprehensive review. Circ. Res..

[30] Weisell RC (2002). Body mass index as an indicator of obesity. Asia Pacific J. Clin. Nutr..

[31] Higgins DM, Moon JC (2014). Review of T1 mapping methods: Comparative effectiveness including reproducibility issues. Curr. Cardiovasc. Imaging Rep..

[32] Krittayaphong RSP, Boonyasirinant T, Nakyen S, Kangkagate C (2004). Gender differences on the left and right ventricular volume, systolic function, and mass assessed by cardiac magnetic resonance imaging. Thai Heart J..

[33] Cerqueira MD (2002). Standardized myocardial segmentation and nomenclature for tomographic imaging of the heart. A statement for healthcare professionals from the Cardiac Imaging Committee of the Council on Clinical Cardiology of the American Heart Association. Int. J. Cardiovasc. Imaging.

[34] Rogers T (2013). Standardization of T1 measurements with MOLLI in differentiation between health and disease—The ConSept study. J. Cardiovasc. Magn. Reson..

[35] Hicks KA (2015). 2014 ACC/AHA key data elements and definitions for cardiovascular endpoint events in clinical trials: A report of the American College of Cardiology/American Heart Association Task Force on Clinical Data Standards (Writing Committee to Develop Cardiovascular Endpoints Data Standards). J. Am. Coll. Cardiol..

