# Supplementary material for: Extracellular volume and left ventricular hypertrophy by cardiac magnetic resonance are independent predictors of cardiovascular outcome in obesity
Source: Sci Rep. 2022 Nov 5;12:18758. doi: 10.1038/s41598-022-23672-1 (PMC9637172; doi:10.1038/s41598-022-23672-1)
Supplement: Supplementary file 1 — Supplementary Information. [file 41598_2022_23672_MOESM1_ESM.pdf]

Supplementary Material

Extracellular volume and left ventricular hypertrophy by cardiac magnetic resonance are independent predictors of cardiovascular outcome in obesity

Panuwat Lertlaksameewilai; Thammarak Songsangjinda, Yodying Kaolawanich, Ahthit Yindeengam; Rungroj Krittayaphong

Supplementary Table 1. Cardiovascular events during follow-up

	**Number of patients**
Death	6 (1.3%)
CV death	1 (0.2%)
Non-CV death	5 (1.1%)
Non-fatal CV event	107 (23.5%)
Heart failure	27 (5.9%)
ACS	6 (1.3%)
STEMI	1 (0.2)
NSTEMI	3 (0.7%)
UA required hospitalization	2 (0.4%)
Revascularization	74 (16.2%)
All CV events	107 (23.5%)

Abbreviations: CV = cardiovascular, ACS = acute coronary syndrome, STEMI = ST-segment elevation myocardial infarction, NSTEMI = non-ST segment elevation myocardial infarction, UA = unstable angina

Supplementary Table 2. Baseline CMR characteristics of the whole population.

Variable	Total
LVEDVI (ml/m^2)	75.6±26.9
LVESVI (ml/m^2)	28.3±25.7
LVMASSI (gram/m^2)	46.0±17.1
LVEF (%)	66.8±15.8
LGE (%)	139 (30.5%)
- Average number of segments with LGE (among patients with LGE)	4.3±3.4
Pattern of LGE	
- One pattern	133
- CAD scar (subendocardial or transmural)	124
- Non cad scar	9
- Midwall	3
- Subepicardial	1
- Patchy	2
- RV insertion	3
- More than one pattern	6
- CAD+midwall	1
- CAD+subepicardial	2
- Midwall+subepicardial	2
- Patchy+RV insertion	1
Wall motion abnormality	146 (32.0%)
- Average number of segments with WMA (among patients with WMA)	7.1±6.0

Abbreviations: LVEDVI = left ventricular end-diastolic volume index, LVESVI = left ventricular end-systolic volume index, LVEF = left ventricular ejection fraction, LGE = late gadolinium enhancement, CAD = coronary artery disesae, RV = right ventricular, WMA = walll motion abnormality
RV insertion scar in this table is not related to hypertrophic cardiomyopathy ((since hypertrophic cardiomyopathy is one of the exclusion criteria)

Supplementary Table 3. Incidence rate of clinical outcomes according to LVH and high ECV

Status	No. of patients	No. of events	100 person-years	Rate per 100 person-years (95% CI)
		**All cause death**		
No LVH	414	5	8.68	0.58 (0.19-1.34)
LVH	42	1	0.83	1.20 (0.03-6.71)
		**CV death**		
No LVH	414	0	8.68	-
LVH	42	1	0.83	1.20 (0.03-6.71)
		**Non-CV death**		
No LVH	414	5	8.68	0.58 (0.19-1.34)
LVH	42	0	0.83	-
		**Composite CV events**		
No LVH	414	87	8.68	10.02 (8.03-12.36)
LVH	42	20	0.83	24.02 (14.72-37.22)
		**HF**		
No LVH	414	22	8.68	2.53 (1.59-3.84)
LVH	42	5	0.83	6.01 (1.96-14.06)
		**ACS**		
No LVH	414	6	8.68	0.69 (0.25-1.50)
LVH	42	1	0.83	1.20 (0.03-6.71)
		**Revascularization**		
No LVH	414	66	13.91	7.60 (5.88-9.67)
LVH	42	15	1.28	18.02 (10.12-29.81)
		**All cause death**		
ECV <30.8%	343	2	7.20	0.28 (0.03-1.00)
ECV ≥30.8%	113	4	2.32	1.73 (0.47-4.41)
		**CV death**		
ECV <30.8%	343	0	7.20	-
ECV ≥30.8%	113	1	2.32	0.43 (0.01-2.40)
		**Non-CV death**		
ECV <30.8%	343	2	7.20	0.28 (0.03-1.00)
ECV ≥30.8%	113	3	2.32	1.30 (0.27-3.78)
		**Composite CV events**		
ECV <30.8%	343	65	7.20	9.03 (6.97-11.51)
ECV ≥30.8%	113	42	2.32	18.13 (13.05-24.47)
		**HF**		
ECV <30.8%	343	14	7.20	1.94 (1.06-3.26)
ECV ≥30.8%	113	13	2.32	5.61 (2.98-9.58)
		**ACS**		
ECV <30.8%	343	4	7.20	0.56 (0.15-1.42)
ECV ≥30.8%	113	3	2.32	1.30 (0.27-3.78)
		**Revascularization**		
ECV <30.8%	343	53	7.20	7.36 (5.51-9.63)
ECV ≥30.8%	113	28	2.32	12.09 (8.02-17.44)

Abbreviations: LVH = left ventricular hypertrophy, ECV = extracellular volume, CI = confidence interval, CV = cardiovascular, HF = heart failure, ACS = acute coronary syndrome

Supplementary Table 4. Univariate and multivariate analyses for factors predicting the composite cardiovascular outcomes

**Factors**	**Univariate analysis**		**Multivariate analysis**	
	**HR (95% CI)**	**p**	**HR (95% CI)**	**p**
Male	1.83 (1.24-2.70)	**0.002**	1.21 (0.78-1.88)	0.404
Age (years)	1.00 (0.98-1.01)	0.665	1.00 (0.98-1.02)	0.850
CAD risk factors				
Smoking	2.64 (1.08-6.50)	**0.034**	1.39 (0.54-3.59)	0.499
Dyslipidemia	0.96 (0.58-1.58)	0.869	0.88 (0.52-1.48)	0.630
Hypertension	1.40 (0.68-2.88)	0.357	0.97 (0.46-2.04)	0.925
Diabetes	1.17 (0.80-1.71)	0.414	1.15 (0.77-1.72)	0.503
History of CAD by coronary angiogram	1.44 (0.96-2.15)	**<0.001**	1.40 (0.86-2.28)	0.268
History of prior ACS	1.95 (1.15-3.33)	**0.014**	1.30 (0.71-2.38)	0.398
eGFR	0.99 (0.98-1.00)	**0.014**	1.00 (0.99-1.01)	0.435
LVEF (%)	0.98 (0.97-0.99)	**<0.001**	1.00 (0.98-1.01)	0.472
LV mass index	1.02 (1.02-1.03)	**<0.001**	1.01 (1.00-1.03)	**0.023**
LGE present	2.97 (2.03-4.34)	**<0.001**	2.14 (1.30-3.54)	**0.003**
ECV ≥30.8%*	2.16 (1.46-3.19)	**<0.001**	1.67 (1.08-2.57)	**0.021**

A *p*-value<0.05 indicates statistical significance (bold)
*Top quartile.
Abbreviations: HR = hazard ratio, CI = confidence interval, CAD = coronary artery disease, ACS = acute coronary syndrome, GFR = glomerular filtration rate, LVEF = left ventricular ejection fraction, LGE = late gadolinium enhancement, ECV = extracellular volume
